# Lower Subjective Socioeconomic Status Is Associated With Increased Risk of Reporting Negative Experiences on Social Media. Findings From the “LifeOnSoMe”-Study

**DOI:** 10.3389/fpubh.2022.873463

**Published:** 2022-06-13

**Authors:** Jens Christoffer Skogen, Tormod Bøe, Turi Reiten Finserås, Børge Sivertsen, Randi Træland Hella, Gunnhild Johnsen Hjetland

**Affiliations:** ^1^Department of Health Promotion, Norwegian Institute of Public Health, Bergen, Norway; ^2^Centre for Evaluation of Public Health Measures, Norwegian Institute of Public Health, Oslo, Norway; ^3^Alcohol and Drug Research Western Norway, Stavanger University Hospital, Stavanger, Norway; ^4^Department of Psychosocial Science, University of Bergen, Bergen, Norway; ^5^Department of Research and Innovation, Helse Fonna HF, Haugesund, Norway; ^6^Department of Mental Health, Norwegian University of Science and Technology, Trondheim, Norway; ^7^Department of Work, Social Services and Housing, Section for Welfare, Bergen Municipality, Bergen, Norway

**Keywords:** social media, adolescence, socioeconomic status, negative experiences, adversity, social gradient, adverse experiences

## Abstract

**Objectives:**

To investigate the association between subjective socioeconomic status (SES) and a) frequency and daily duration of social media use, and b) self-reported negative experiences on social media platforms.

**Methods:**

The present study is based on the cross-sectional school-based “LifeOnSoMe”-study (*N* = 3,415) recruiting high school students aged 16 years or more in Bergen, Norway. Subjective SES was the independent variable and measured by perceived family affluence. The dependent variables included self-reported amount of social media use, and eight different types of negative experiences on social media. Self-reported age, gender, country of birth and type study were used as covariates. Statistical analyses included multinomial logistic regression and negative binomial regression models.

**Results:**

For amount of social media use, we only found relatively weak and inconsistent associations with SES. In contrast, the associations between SES and separate variables gauging negative experiences were robust in crude models as well as in models adjusted for age and gender. The number of different negative experiences were increased by 1.25 times for those with low and by 1.10 times for those with medium socioeconomic status, compared to those with high socioeconomic status in fully adjusted models. For composite measures of “negative acts and exclusion” and “unwanted attention from others,” the difference between low and high SES was equivalent to a small-to-moderate effect size even after adjustments for age, gender, country of birth, type of study and amount of social media use.

**Conclusions:**

In the present study, we found consistent and strong support for an association between SES and negative experiences on social media even after adjustments for age, gender, country of birth, type of study, and amount of social media use. The potential link between SES and negative experiences on social media as reported in this study is likely to have a public health impact. As the reported findings are novel, they need to be replicated in forthcoming studies based on other study populations. Future research should also focus on other aspects of SES and negative experiences, as well as endeavor to investigate potential longitudinal associations.

## Introduction

Over the last decades social media has permeated most parts of society, and the most widespread social media platforms such as Snapchat, Instagram, and Facebook have up to 2.9 billion active users (1). Social media use is particularly ubiquitous among adolescents, and nearly half of US adolescents report using social media “almost constantly” (2). Norwegian surveys have shown that well over 90 % of adolescents are on at least one social media platform and almost half spend 2–3 hours or more on social media every day (3).

Concerns have been raised about the effects of social media use on adolescents' mental health, and social media use has been associated with depression, anxiety, and reduced well-being (4). The results are however, mixed with other studies reporting no, or even positive, associations. Overall, meta-analyses show a negative, but very small effect of the amount of social media use on well-being (5), and some have questioned the practical importance of these effects (6). Consequently, researchers have called for studies on specific aspects of social media use, rather than merely the amount of use (7, 8).

Furthermore, previous studies have largely aggregated social media effects on large groups of adolescents. It seems, however, that the use of, experiences with and potential effect of social media may differ across individuals and subgroups. Valkenburg, et al. (9) showed that 20 % of adolescents experienced negative effects of passive social media use (i.e., passively scrolling through social media content), while 80 % did not. A recent study demonstrated that 10 % experienced negative effects of passive social media use on their affective well-being, while 44 % felt neither better nor worse and 46 % felt better (10). These findings are in line with the “differential susceptibility to media effects model” by Valkenburg & Peter (11), which rejects universal media effects and posits that the effect of media depends on individual variables and social context. A more fruitful endeavor, then, would be to identify those individuals at risk of being affected negatively by their social media use. There has, however, been a lack of attention to contextual risk factors in terms of susceptibility to negative effects of social media use (4).

Socioeconomic status (SES) is one potential social-context variable which may play a role in adolescents' experiences with social media use. Adolescent SES may be defined according to objective indicators such as their parent's education level and occupation or their household income, or as their subjective socioeconomic status with reference to the adolescent's perception of his or her place in the social status order (12, 13). Studies have shown a social gradient in screen time (14, 15), with more time spent on screen-based activities among people with low SES compared to high SES. One study of children and adolescents found a negative association between SES and social media addiction (16). Furthermore, it is more common to have access to media devices in the bedroom among adolescents from low-income compared to high-income families (17).

One concern with social media is the risk of negative online experiences. Adolescents have reported being exposed to harmful content, such as ways to physically self-harm or commit suicide, hate messages, and violent images (18), and some are targets of bullying (19), cyber victimization (20), and sexual harassment and solicitation (21). One third of European 15–16 year olds have reported having negative online experiences that made them feel upset, scared, or uncomfortable (18), but the prevalence of negative online experiences vary considerably (22).

For negative/adverse life events in general, there is a clear relationship with childhood socioeconomic status (23). With this knowledge as a backdrop, some studies have investigated the relationship between negative online experiences and SES but so far, the results have been mixed. One study found that vulnerability to online grooming was associated with low SES (24), while other studies have found no direct association between SES and online risks (25, 26). One study found that having at least one parent with a university degree (as a proxy for high SES) was associated with lower likelihood of being victim or perpetrator of cyber victimization (27). Others have found no association between SES and cyber victimization (28). Beyond these examples, research on SES and negative experiences on social media remain scarce.

Identifying individuals at risk for negative effects of social media use or negative experiences on social media is important to be able to reduce harm from social media. Since there might be a parallel link between SES and negative experiences on social media as the one observed in general, an investigation of this is warranted. Consequently, the aim of this exploratory study is to investigate the association between subjective SES (based on subjective “perceived family affluence”) and (a) frequency and daily duration of social media use, and (b) self-reported negative experiences on social media platforms, while accounting for some potentially important.

## Materials and Methods

### Sample

The “LifeOnSoMe”-study sample consists of two school-based data collections during September-October 2020 and June-September 2021 recruiting high school students aged 16 years or more in the municipality of Bergen, Norway. In 2020, the participation rate was 53% while the participation rate was 35.4% in 2021. The total number of participants eligible for the present study was *N* = 3,415 with a mean age of 17.3 (standard deviation 1.0), and 44% were boys. Both data collections were web-based, and the potential participants received a survey-specific web address containing written online information about the study as well as the possibility to consent to participate. The study was approved by the regional ethics committee and is in agreement with the General Data Protection Regulation (See also “Ethics” below for more information).

### Study Variables

#### Age and Gender

Age and gender were registered by self-report. Gender included a non-binary option, but only 37 participants ticked this option. Due to the small number, they were excluded from further analyses in the present study.

#### Country of Birth and Type of Study

The participants could indicate whether they were born in Norway or in another country. They were also asked about the *type of study* they attended, where the options were “general studies,” with specialization in general studies as the major specific educational programme, and “vocational studies” aimed at specific vocations such as building and construction, electrical engineering, and computer technology.

#### Subjective Socioeconomic Status

The participants could indicate their subjective socioeconomic status (S-SES) by answering the question “How well off do you consider your own family to be compared to others?,” ranging from 0 (“Very poor”) to 10 (“Very well off”). The distribution of the S-SES variable was right-skewed with mean of 7.2 (standard deviation 1.8). For the purposes of the present study, a tripartite variable was created differentiating between low SES (scores 0–4; 6.3%), medium SES (scores 5–7; 52%), and high SES (scores 8–10; 41.7%).

#### Amount of Social Media Use

Two questions regarding amount of social media use were asked. The wording of the first question was “How often do you use social media?” with eight response options: “Almost never,” “Many times a week but less than weekly,” “1–2 times a week,” “3–4 times a week,” “5–6 times a week,” “Daily,” “Many times a day” and “Almost constantly.” For the purposes of the present study, we differentiated between “Daily or less” (24%), “Many times a day” (49.4%) and “Almost constantly” (26.6%). The wording of the second question was “The days you use social media, approximately how much time do you spend per day?,” with five response options: “<30 min”, “between 30 min and 1 h”, “1–2 h”, “2–4 h” and “>5 h”. In the present study, we differentiated between “<2 h” (29.5%), “2–4 h” (38.1%), 4–5 h” (18%) and “>5 h” (14.4%).

#### Negative Experiences on Social Media

Eight statements regarding negative experiences on social media were asked, each with five potential responses: “Never,” “Seldom,” “Sometimes,” “Often,” and “Very often.” The statements are derived from analyses of focus group interviews of adolescents regarding social media use and mental health and well-being (29). The statements were:

Variable 1: “I get unwanted attention from strangers”
Variable 2: “Others share pictures/videos against my will”
Variable 3: “I receive unwanted nude pictures/sexualised content”
Variable 4: “I am asked to send nude pictures/sexualised content”
Variable 5: “I get negative comments on my posts”
Variable 6: “I receive unpleasant/hurtful messages”
Variable 7: “Others say/post negative things about me”
Variable 8: “I feel excluded from groups/chats”

The variables 1, 3, and 4 were combined as a composite measure of “Unwanted attention from others” and the remaining five variables were combined as a composite measure of “Negative acts and exclusion.” Lastly, we also estimated the number of endorsed items (i.e., more than “never”) ranging from 0 (23.1%) to 8 (7.4%). This count variable was named “Number of different negative experiences.”

### Statistical Analyses

**Figure 1:**
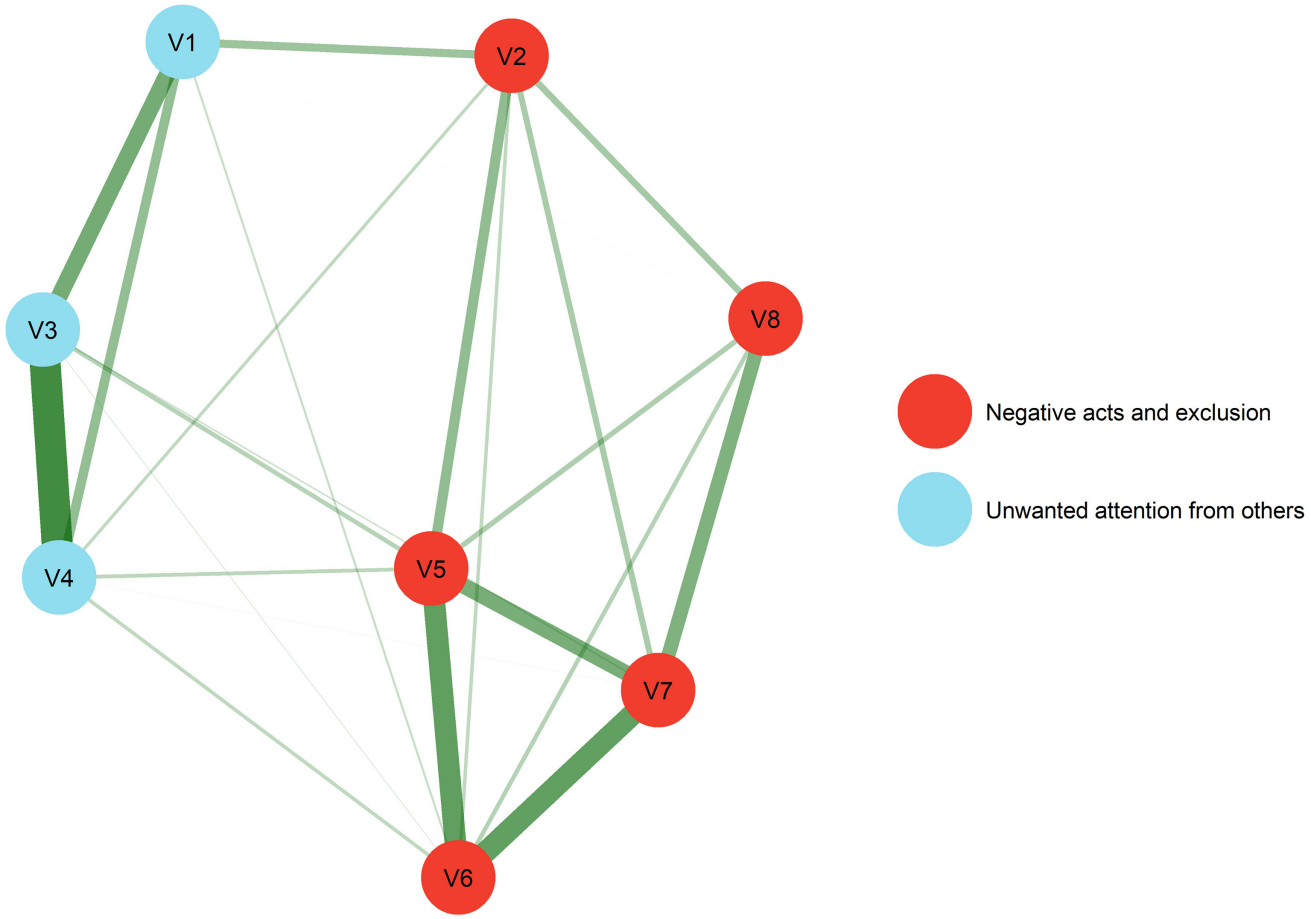
Results from exploratory graph analysis. Unwanted attention from strangers (V1) Others share pictures/videos (V2) Receive unwanted nude pictures/sexualised content (V3) Asked to send nude pictures/sexualised content (V4) Negative comments (V5) Unpleasant/hurtful messages (V6) Negative talk from others (V7) Excluded from groups/chats (V8).

First, exploratory graph analysis and confirmatory factor analysis was employed to determine the dimensionality of the eight statements regarding negative experiences on social media. Figure 1 depicts the results from the exploratory graph analysis and estimates from the following confirmatory factor analysis of the suggested dimensions are presented in the results section. Next, results from descriptive analyses of the included variables are presented across subjective socioeconomic status (Table 1), using mean and standard deviation for continuous data and frequency and proportion for categorical data. Due to privacy concerns as a consequence of small numbers in some of the response categories on the eight negative experiences, we only differentiate between “Never,” “Seldom,” and “Sometimes or more” in Table 1.

**Table 1:** Description of included variables across subjective socioeconomic status.

**Characteristic**	**Low (0–4), *N* = 216^**a**^**	**Medium (5–7), *N* = 1,778^**a**^**	**High (8–10), *N* = 1,421^**a**^**	***p*-value^**b**^**
**Age**	17.60 (1.16)	17.27 (1.00)	17.24 (0.94)	**<0.001**
**Gender**				**<0.001**
Boys	67 (31%)	705 (40%)	723 (51%)	
Girls	149 (69%)	1,073 (60%)	698 (49%)	
**Country of birth**				**0.028**
Norway	189 (88%)	1,635 (92%)	1,277 (90%)	
Other country	27 (12%)	143 (8.0%)	144 (10%)	
**Type of study**				0.093
General studies	149 (69%)	1,328 (75%)	1,078 (76%)	
Vocational studies	67 (31%)	450 (25%)	343 (24%)	
**Frequency of social media use**				0.4
Daily or less	46 (21%)	424 (24%)	349 (25%)	
Many times a day	101 (47%)	888 (50%)	698 (49%)	
Almost constantly	69 (32%)	466 (26%)	374 (26%)	
**Daily duration of social media use**				**0.030**
<2 hours	49 (23%)	520 (29%)	438 (31%)	
2–4 hours	77 (36%)	667 (38%)	556 (39%)	
4–5 hours	48 (22%)	331 (19%)	237 (17%)	
>5 hours	42 (19%)	260 (15%)	190 (13%)	
**Unwanted attention from strangers (V1)**				**<0.001**
Never	51 (25%)	541 (32%)	494 (37%)	
Seldom	79 (38%)	565 (33%)	434 (33%)	
Sometimes or more	77 (37%)	590 (35%)	399 (30%)	
**Others share pictures/videos (V2)**				**<0.001**
Never	106 (51%)	966 (57%)	861 (65%)	
Seldom	78 (38%)	569 (33%)	345 (26%)	
Sometimes or more	23 (11%)	167 (9.8%)	127 (9.5%)	
**Receive unwanted nude pictures/sexualised content (V3)**				**0.006**
Never	87 (42%)	906 (53%)	749 (56%)	
Seldom	58 (28%)	383 (23%)	285 (21%)	
Sometimes or more	61 (30%)	412 (24%)	298 (22%)	
**Asked to send nude pictures/sexualised content (V4)**				**<0.001**
Never	105 (51%)	993 (58%)	852 (64%)	
Seldom	39 (19%)	344 (20%)	268 (20%)	
Sometimes or more	61 (30%)	365 (21%)	212 (16%)	
**Negative comments (V5)**				**<0.001**
Never	153 (74%)	1,422 (84%)	1,155 (87%)	
Seldom	36 (17%)	199 (12%)	114 (8.6%)	
Sometimes or more	18 (8.7%)	76 (4.5%)	59 (4.4%)	
**Unpleasant/hurtful messages (V6)**				**<0.001**
Never	128 (62%)	1,238 (73%)	1,053 (79%)	
Seldom	47 (23%)	316 (19%)	183 (14%)	
Sometimes or more	32 (15%)	146 (8.6%)	95 (7.1%)	
**Negative talk from others (V7)**				**<0.001**
Never	134 (65%)	1,238 (73%)	1,037 (78%)	
Seldom	45 (22%)	314 (19%)	178 (13%)	
Sometimes or more	27 (13%)	142 (8.4%)	111 (8.4%)	
**Excluded from groups/chats (V8)**				**<0.001**
Never	117 (57%)	1,013 (60%)	874 (66%)	
Seldom	50 (24%)	453 (27%)	289 (22%)	
Sometimes or more	39 (19%)	234 (14%)	162 (12%)	
**Negative acts and exclusion (mean)**	1.56 (0.68)	1.42 (0.55)	1.36 (0.58)	**<0.001**
**Unwanted attention from others (mean)**	2.09 (0.99)	1.89 (0.89)	1.79 (0.86)	**<0.001**
**Number of different negative experiences (mean)**	3.56 (2.62)	2.97 (2.51)	2.50 (2.42)	**<0.001**

*N = 3,415. ^a^Mean (SD); n (%); ^b^Kruskal-Wallis rank sum test; Pearson's Chi-squared test*.

*Bold indicates statistical significant difference*.

*Missing information on variables ranging from n = 192 (5.6%) to n = 167 (4.9%)*.

The association between socioeconomic status and amount (frequency and duration) of social media use was estimated using multinomial logistic regression with socioeconomic status as the main predictor. The base level was “daily or less” for frequency of social media use, and “<2 h” for daily duration of use, and the reference category for socioeconomic status was high SES. The results, presented as relative risk ratios (RRR; 95% confidence intervals) from crude models and models adjusted for age, gender, country of birth and type of study are presented in Table 2.

**Table 2:** Results from multinomial logistic regression for frequency and daily duration of social media use.

**Variable**	**Crude, relative risk ratio (95% CI)**	***p*-value**	**Adjusted, relative risk ratio (95% CI)**	***p*-value**
**Frequency of social media use**	Base category (daily or less)	N/A	Base category (daily or less)	N/A
Many times a day, low SES (0–4)	1.10 (0.76–1.58)	0.613	1.07 (0.74–1.55)	0.711
Almost constantly, low SES (0–4)	**1.40 (1.01–1.95)**	**0.045**	1.21 (0.85–1.74)	0.296
Many times a day, medium SES (5–7)	1.05 (0.89–1.28)	0.647	0.99 (0.81–1.21)	0.923
Almost constantly, medium SES (5–7)	1.03 (0.81–1.30)	0.833	0.93 (0.73–1.19)	0.566
**Daily duration of social media use**	Base category (<2 h)	N/A	Base category (<2 h)	N/A
2–3 hours, low SES (0–4)	1.24 (0.86–1.78)	0.250	1.09 (0.76–1.56)	0.642
4–5 hours, low SES (0–4)	**1.81 (1.27–2.58)**	**0.001**	1.44 (1.00–2.07)	0.052
>5 hours, low SES (0–4)	**1.98 (1.31–2.98)**	**0.001**	**1.52 (1.01–2.30)**	**0.046**
2–3 hours, medium SES (5–7)	1.01 (0.85–1.20)	0.904	0.94 (0.79–1.12)	0.501
4–5 hours, medium SES (5–7)	1.18 (0.96–1.45)	0.122	1.04 (0.84–1.28)	0.726
>5 hours, medium SES (5–7)	1.15 (0.94–1.42)	0.176	1.02 (0.83–1.25)	0.848

*Subjective socioeconomic status as independent variable [reference group; high SES (8–10)] use of social media as dependent variable. Crude associations and adjusted for age and gender, country of birth and type of study. N = 3,415*.

*95% CI, 95% Confidence interval; SES, Socioeconomic status. Adjusted: Adjusted for age and gender, country of birth and type of study*.

*Bold indicates significant estimates*.

*Multiple-imputation estimates based on multinomial logistic regression models while accounting for clustering (school and grade level) of data*.

**Figure 2:**
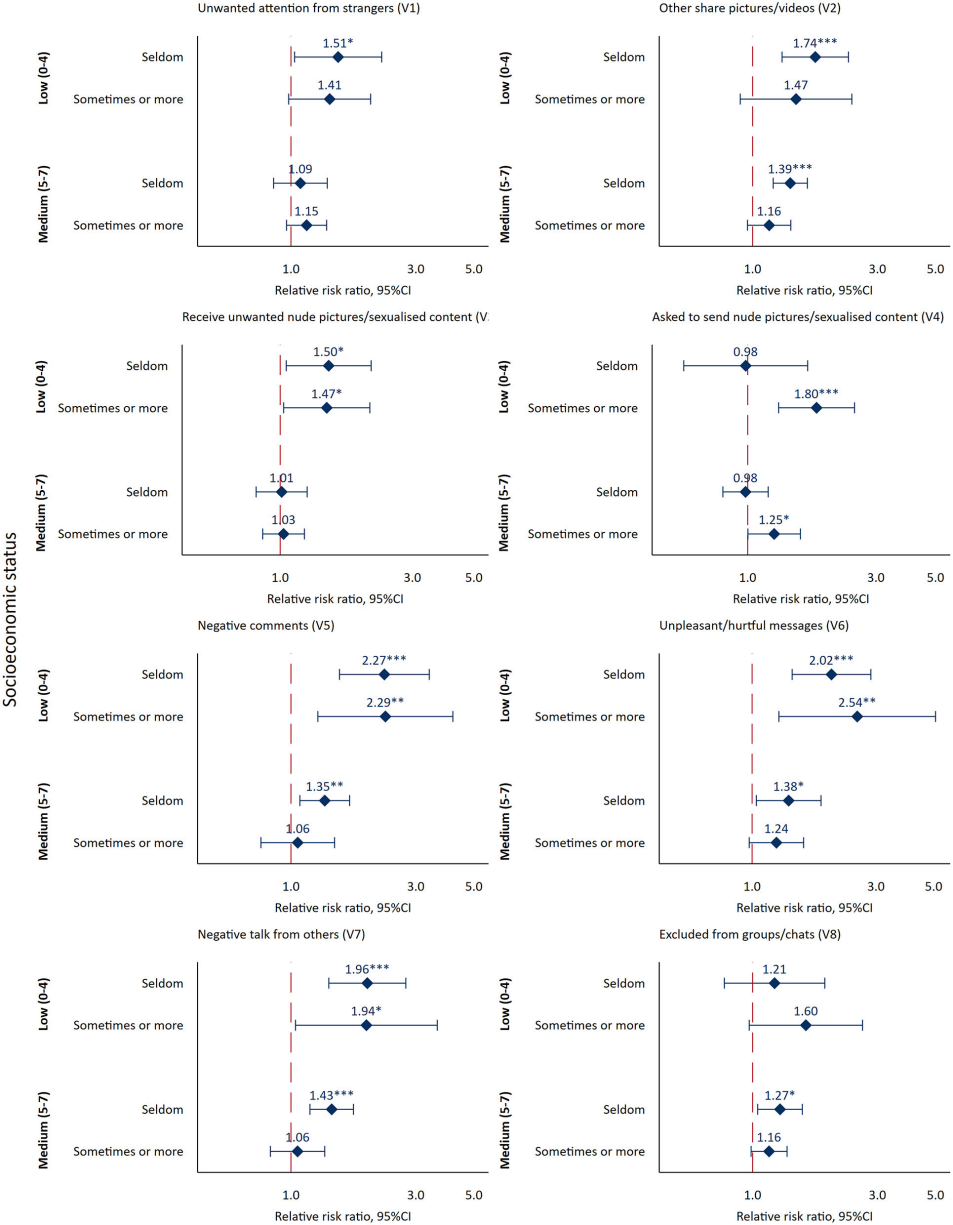
Results from multinomial logistic regression across negative experiences on social media. Subjective socioeconomic status as independent variable [reference group: high socioeconomic status (SES; 8–10)] and negative experiences (base category: never) on social media as dependent variable. Adjusted for age and gender. Estimates based on multiple imputation while accounting for school-level clustering. Base category: Never; Reference group: High SES (8–10); *p < 0.05, **p < 0.01, ***p < 0.001. Red dotted line indicates no difference in relative risk.

The association between socioeconomic status and each of the eight negative experiences were also estimated using multinomial logistic regression (base level “never” and “high SES” as reference), and the results from age and gender-adjusted models are presented in Figure 2. Due to small numbers in some of the response categories, a tripartite variable was created differentiating between “Never,” “Seldom,” and “Sometimes or more” for each of the variables gauging negative experiences. Additional results from multinomial logistic regression estimating the crude association between socioeconomic status and negative experiences on social media are presented in Supplementary Table 1. The association between socioeconomic status and number of different negative experiences endorsed were estimated using negative binomial regression models (“high SES” as reference) as they are suitable for count data and able to accommodate for overdispersion. For the association with number of different negative experiences, a crude model, a model adjusted for age, gender, country of birth and type of study, as well as a fully adjusted model adjusting for age, gender, country of birth, type of study and amount of social media use was estimated, and the results are presented in Table 3 as incidence rate ratios (IRR; 95% confidence intervals). The association between socioeconomic status and the composite scores (“Negative comments and exclusion” and “Unwanted attention from others”) were estimated using multilevel regression models, where the composite scores were entered as continuous dependent variables in separate regression models. To aid interpretation the composite scores were z-scored (mean 0 and standard deviation 1). For both composite scores, a crude model, a model adjusted for age and gender, country of birth and type of study, and a model adjusted for age, gender, country of birth, type of study and frequency and duration of social media use was estimated. The results from these analyses are presented in Table 4. The response distribution of number of different negative experiences endorsed and the two composite scores across socioeconomic status is presented as ridge plots in the Supplementary Material (see Supplementary Figure 1).

**Table 3:** Association between subjective socioeconomic status and number of different negative experiences on social media.

	**Crude**		**Adjusted for age, gender, country of birth, and type of study**		**Fully adjusted**	
	**IRR (95% CI)**	***p*-value**	**IRR (95%CI)**	***p*-value**	**IRR (95%CI)**	***p*-value**
Low SES (0–4)	**1.36** **(1.19–1.56)**	**<0.001**	**1.28** **(1.12–1.47)**	**<0.001**	**1.25** **(1.09–1.43)**	**0.001**
Medium SES (5–7)	**1.15** **(1.07–1.23)**	**<0.001**	**1.10** **(1.02–1.18)**	**0.011**	**1.10** **(1.02–1.18)**	**0.011**
High SES (8–10)	Ref.	–	Ref.	–	Ref.	–

*N = 3,415. 95% CI, 95% Confidence Interval; IRR, Incidence Rate Ratio; SES, Socioeconomic status*. *Bold indicates significant estimates. Fully adjusted: adjusted for age, gender, country of birth, type of study, frequency of social media use and daily duration of social media use*. *Multiple-imputation estimates based on negative binomial regression models accounting for the clustering (school level) of data*.

**Table 4:** Association between subjective socioeconomic status and mean level of negative experiences on social media, I) negative acts and exclusion and II) unwanted attention from others.

	**Crude**		**Adjusted for age, gender, country of birth, and type of study**		**Fully adjusted**	
**Variables**	**Beta (95% CI)**	***p*-value**	**Beta (95% CI)**	***p*-value**	**Beta (95% CI)**	***p*-value**
**I) Negative acts and exclusion**						
Low SES (0–4)	**0.33** **(0.19–0.48)**	**<0.001**	**0.31** **(0.16–0.46)**	**<0.001**	**0.29** **(0.15–0.44)**	**<0.001**
Medium SES (5–7)	**0.09** **(0.02–0.16)**	**0.012**	**0.08** **(0.01–0.15)**	**0.035**	**0.08** **(0.01–0.15)**	**0.030**
High SES (8–10)	Ref.	–	Ref.	–	Ref.	–
**II) Unwanted attention from others**						
Low SES (0–4)	**0.32** **(0.18–0.47)**	**<0.001**	**0.20** **(0.06–0.34)**	**0.005**	**0.18** **(0.04–0.32)**	**0.012**
Medium SES (5–7)	**0.10** **(0.03–0.17)**	**0.005**	0.04 (−0.03–0.10)	0.321	0.04 (−0.03–0.11)	0.281
High SES (8–10)	Ref.	–	Ref.	–	Ref.	–

*Dependent variables entered as Z-scored continuous variables. N = 3,415*.

*95% CI, 95% Confidence Interval; SES, Socioeconomic status. Bold indicates significant estimates*.

*Fully adjusted: adjusted for age, gender, country of birth, type of study, frequency of social media use, and daily duration of social media use*.

*Multiple-imputation estimates based on multilevel regression models accounting for the clustering (school and grade level) of data*.

Exploratory graph analysis and confirmatory factor analysis were carried out in R (30) using the packages “EGAnet” (31) and “lavaan” (32). For the confirmatory factor analyses the diagonally weighted least squares (DWLS) estimator was employed as the data was non-normally distributed ordered data (33, 34). Data handling and all other analyses were performed in Stata version 15 (35). Due to missing on the eight negative experiences on social media variables, 50 datasets were with imputed data using multiple imputation under the assumption of at least missing at random (36). For all analytical statistical tests and regression models, estimates from the imputed datasets are presented (Tables 2–4; Figure 2; Supplementary Table 1) while accounting for clustering of the data. The results from analyses employing pairwise deletion (data not shown) were similar to those presented.

### Ethics

The data collections were approved by the Regional Ethics Committee (REK) in Norway (REK#65611) and conducted according to the guidelines of the Declaration of Helsinki. All participants were informed about the general purpose of the study and participants gave their informed consent upon participation. They were also informed about the opportunity to withdraw from the study even after initial participation. As all participants invited were 16 years or older, they were deemed competent to consent on their own behalf, and additional consent from parents or guardians was not called for.

## Results

### Dimensionality of Negative Experiences on Social Media

Exploratory graph analysis (EGA) indicated two factors, one factor with five items and a second factor with three items (Figure 1). The same solution was replicated 500 times, suggesting perfect structural consistency with all items replicating in their empirical dimension. A confirmatory factor analysis with two correlated factors yielded satisfactory fit (RMSEA: 0.060 (90% CI 0.053, 0.067); CFI: 0.992; TLI: 0.989; SRMR: 0.031). The correlation between the two factors were 0.69, and factor loadings ranged from 0.69 to 0.92 (mean 0.84). The first factor encompassed the variables “Others share pictures/videos (V2),” “Negative comments (V5),” “Unpleasant/hurtful messages (V6),” “Negative talk from others (V7),” and “Excluded from groups/chats (V8),” and was named “Factor 1: Negative acts and exclusion.” The second factor encompassed “Unwanted attention from strangers (V1),” “Receive unwanted nude pictures/sexualised content (V3),” and “Asked to send nude pictures/sexualised content (V4),” and was named “Factor 2: Unwanted attention from others.”

### Descriptive Analyses Across Socioeconomic Status

Those with low SES were somewhat older compared to the other groups (p <0.001), and more likely to be girls (*p* < 0.001; Table 1). The low SES group was also more likely to be born in another country than Norway (*p* = 0.028). There was no difference in type of study or frequency of social media use across SES-groups, but those with lower SES indicated a higher daily duration of social media use (*p* = 0.030). In relation to negative experiences on social media, lower SES was associated with higher frequency for all included variables (*p*-values ranging from 0.006 to <0.001). Overall, there was a tendency to report a higher frequency of negative experiences on social media with decreasing subjective socioeconomic status. The mean number of endorsed negative experiences was 3.6 for those with low SES, 3.0 for those with medium SES, and 2.5 for those with high SES.

### Associations Between Socioeconomic Status and Amount of Social Media Use

The results from adjusted multinomial logistic regression models with frequency of social media use as the dependent variable indicated no association with subjective socioeconomic status (see Table 2). For daily duration of social media use, however, low SES was associated with increased relative risk for a higher duration of social media use (4–5 hours and >5 hours) compared to high SES. In the adjusted model, only the association between low SES and >5 hours or more was statistically significant [relative risk ratio (RRR): 1.52].

### Associations Between Socioeconomic Status and Negative Experiences on Social Media

The results from separate age- and gender-adjusted multinomial logistic regressions for each item regarding negative experiences on social media across subjective socioeconomic status are presented in Figure 2. For “Unwanted attention from strangers (V1),” low SES was associated with increased relative risk for the response category “seldom” compared to high SES. For “Others share pictures/videos (V2),” both low and medium SES was associated with increased relative risk for the category “seldom” compared to high SES. For “Receive unwanted nude pictures/sexualised content (V3)” low SES was associated with increased relative risk for the response categories “seldom” and “sometimes or more.” For “Asked to send nude pictures/sexualised content (V4),” increased relative risk for low and medium SES was observed for the category “sometimes or more.” Low SES was associated with increased relative risk for “Negative comments (V5)” for both response categories (“seldom” and “sometimes or more”) compared to high SES, while medium SES was associated with increased relative risk for the “seldom” response category. The same pattern was seen for “Unpleasant/hurtful messages (V6),” with increased relative risk for low SES compared to high SES across both response categories, and increased risk for medium SES for the response category “seldom.” There was increased relative risk for “Negative talk from others (V7)” for both “seldom” and “sometimes or more” for low SES and increased relative risk for “seldom” for medium SES compared to high SES. Lastly, for “Excluded from groups/chats (V8),” medium SES was associated with increased risk for the response category “seldom” compared to high SES. Both crude and age- and gender adjusted multinomial logistic regression models are presented in Supplementary Table 1. Overall, non-substantial changes to the point estimates were observed when comparing the crude estimates with the age- and gender-adjusted ones, but number of significant associations was reduced from 21 to 17.

Socioeconomic status was negatively associated with number of different negative experiences on social media (Table 3). Compared to high socioeconomic status, the crude incidence rate ratios was 1.36 and 1.15 for low and medium levels, respectively. In the fully adjusted model, the corresponding IRRs were 1.25 and 1.10.

Low socioeconomic status was associated with increased mean score on both “negative acts and exclusion” (0.33 standard deviation difference in the crude model) and “unwanted attention from others” (0.32 standard deviation) compared to high socioeconomic status (Table 4). For “negative acts and exclusion,” only small changes in estimates across adjustment levels were observed. For “unwanted attention from others,” the change in estimates were more marked with a 44% reduction in the coefficient. Medium socioeconomic status was associated with increased mean score on both “negative acts and exclusion” and “unwanted attention from others” (standard deviations 0.09 and 0.10, respectively) compared to high socioeconomic status in crude models. For “negative acts and exclusion” only small changes in estimates across adjustment levels were observed. For “unwanted attention from others,” however, the change in estimates were marked (60%) and the association was rendered non-significant in the fully adjusted model.

## Discussion

### Main Findings

Using data from a school-based study of older adolescents, we investigated the potential association between socioeconomic status and (a) amount of social media use and (b) negative experiences on social media. For amount of social media use, we found inconsistent associations, and only the association between low SES and >5 hours or more daily use was statistically significant after adjusting for age, gender, country of birth and type of study. In contrast, the associations between socioeconomic status and separate variables gauging negative experiences were robust in crude models as well as in models adjusted for age and gender. The number of different negative experiences was increased by 1.25 times for those with low and by 1.10 times for those with medium socioeconomic status, compared to those with high socioeconomic status in fully adjusted models. For the composite measures of “negative acts and exclusion” and “unwanted attention from others,” the difference between low and high SES was equivalent to a small-to-moderate effect size even after adjustments for age, gender, country of birth, type of study and amount of social media use.

### Interpretation and Public Health Implications

The present results demonstrate the importance of assessing specific aspects of social media use beyond the mere amount. We found no association between socioeconomic status and frequency of use, and only the longest duration of social media use was statistically different when comparing low and high socioeconomic status. For negative experiences on social media, however, we found clear indications of a social gradient robust for adjustments.

Our results cannot provide an explanation for the observed association between socioeconomic status and negative experiences on social media, and future studies should also seek to assess potential causality and as well as potential underlying mechanisms. The association may be related to a social differential in the type of social media platforms adolescents' use, who they interact with, how they interact with others, or how much they expose themselves in both private and public posts on social media. Higher levels of self-presentation on social media have for instance been found to be associated with higher levels of mental distress among adolescents (37), and it can be speculated that those who expose themselves a lot on social media also experience more negative experiences such as unwanted sexual attention. One study found that receiving negative feedback on social media was predicted by adolescents' tendency to engage in risky online self-presentation, and also by their tendency to engage in social exploration (38). We are, however, not aware of any published papers specifically investigating the role of SES to online self-presentation and negative experiences. Even so, a recent pre-publication (pending review) reported that there seems to be an association between SES and different levels focus of self-presentation on social media, although the results were not robust to adjustments (39).

It is well-documented that there is a social gradient in exposure to adverse life events, such as divorce, deaths in close family and maltreatment while growing up. The increased risk of an array of adverse exposures is often understood as a clustering effect since those in the lower tail of the socioeconomic gradient are disproportionally affected by this in a cumulative manner during the life course (40, 41). Furthermore, adverse life events are associated with negative short-term and long-term consequences. This includes poorer physical and mental health, lower educational attainment and stunted vocational opportunities (42–44). There is also strong evidence pointing toward a social gradient in peer-victimization such as being exposed to bullying (45, 46), with substantial negative ramifications to health and well-being into adulthood of the victim (47, 48).

With the advent of social media and subsequent widespread use, several studies have focused on cyberbullying. In summary, research have indicated that cyberbullying can be considered an extension (although with certain specific characteristics) of face-to-face bullying (8), and that social media should be considered a “new tool to harm victims already bullied by traditional means” (49). Although our study did not measure (cyber) bullying per se, the included items related to negative experiences on social media can be considered cardinal features of behavior that constitute bullying, such as negative verbal actions, negative non-verbal actions, and social exclusion (50). Admittedly, we are not able to gauge fundamental characteristics of (cyber) bullying such as power-imbalance and whether or not the behaviors are deliberately hurtful from the perspective of the acting individual (51). Despite this, we believe that our measures of negative experiences on social media may be important in relation to the individuals' sense of belongingness in their day-to-day interactions on social media and their mental health and well-being in general. Also, the included measures are closely linked to the broader phenomenon of cyber victimization, which is frequently linked to internalizing and externalizing problems among adolescents (52). Although the associations observed can be considered small-to-medium with regards to effect size, we believe that our findings warrant further investigation, especially since it can be argued that a social gradient in exposure to negative experiences on social media probably is in addition to the “accumulation of disadvantages” generally observed outside of social media.

From a public health perspective, it is therefore important to ascertain the potential disproportional exposure to negative experiences on social media related to differential socioeconomic status together with other areas where the social gradient is more established. As in other areas where we see a social gradient, modification of the root cause (social inequity) is insurmountable without major societal changes—but some universal preventive measures may be helpful in relation to negative experiences on social media. First, increased awareness regarding potentially harmful behaviors and interactions on social media needs to be established among children/adolescents, parents and other adults such as teachers. Second, parents and teachers need to become more involved in the social media part of children/adolescents' lives. Third, increased knowledge about how social media interactions are similar and dissimilar to face-to-face interactions may be helpful to avoid potential harmful behaviors that are not intended.

### Strengths and Limitations

The present study holds several strengths. First, data collection is recent and specifically focus on different aspects of social media use among adolescents. Second, the number of participants was large, enabling meaningful investigations into potential confounding factors related to the associations between socioeconomic status and amount of social media use, and negative experiences on social media. Third, this is the first study, to our knowledge, that have investigated the potential association between socioeconomic status and a range of different negative experiences on social media. Despite these strengths, the study also have some notable limitations. First, the participation rate was rather low, especially for the data collection in 2021. The low participation rate can probably partly be explained by measures and restrictions posed on schools and pupils in relation to the ongoing COVID-19 pandemic. For 2021 specifically, the start of the data collection coincided with a school-staff strike leading to severely disrupted services and closings in several of the schools in the catchment area. It is likely this further curbed participation rates in the affected schools. Bias due to low participation rates are, however, more likely to affect descriptive epidemiology focusing on prevalence as compared to analytical epidemiology focusing on associations (53). In support of this notion, the overall findings presented in the present paper were similar when analyzing data from 2020 and 2021 separately (data not shown). We therefore consider our findings to be valid despite the relatively low participation rate. A second limitation is that the data analyzed was cross-sectional, which negates the possibility to make clear statements about timing and potential causality between the included factors. Third, we only included one general subjective measure on socioeconomic status, and inclusion of other objective measures of socioeconomic status such as parental education may lead to other findings. The use of objective indicators, such as income, education, and occupation are frequent in the research literature, but they are more likely to gauge specific aspects of socioeconomic status. In adult populations, however, subjective indicators been shown to be more strongly associated with health outcomes compared to objective indicators of socioeconomic status (54). A meta-analytical study also found that subjective socioeconomic status is associated with a range of important health outcomes (13). Subjective socioeconomic status is, however, a widely used general proxy for socioeconomic status and have been shown to be reliably associated with objective indicators such as household income, and parental work status and education levels in studies of Norwegian adolescents (55, 56). Fourth, the data collection is restricted to one geographic area, and the findings may therefore not be generalizable to other areas. Fifth, in this exploratory study, we only included a few potential covariates in the analyses, and potential important residual confounding cannot be ruled out.

## Conclusions

In the present study of older adolescents examining the association between one aspect of socioeconomic status and amount of social media use and negative experiences on social media, we found consistent and strong support for an association between socioeconomic status and negative experiences even after adjustments for age, gender, country of birth, type of study and amount of social media use. In contrast, the evidence of an association between socioeconomic status and amount of social media use was relatively weak and inconsistent. This supports the rising notion that there is a need to move beyond mere measures of duration or frequency of social media use, and focus more on other aspects such as experiences and behavior on social media platforms. The potential link between socioeconomic status and negative experiences on social media as reported in this study is likely to have a public health impact for those disproportionally affected. As the reported findings are novel, they need to be replicated in forthcoming studies based on other study populations. Future research should also focus on other aspects of socioeconomic status and negative experiences, as well as endeavor to investigate potential longitudinal associations.

## Data Availability Statement

The data analyzed in this study is subject to the following licenses/restrictions. The Norwegian Health research legislation and the Norwegian Ethics Committee require explicit consent from the participants to transfer health research data outside of Norway. For the data employed in the present study, ethics approval was also contingent on storing the research data on secure storage facilities located at the Norwegian Institute of Public Health, which prevents us from providing the data as Supplementary Material or to transfer it to data repositories. Requests to access these datasets should be directed to JS, jens.christoffer.skogen@fhi.no.

## Ethics Statement

The studies involving human participants were reviewed and approved by Regional Ethics Committee of Norway. Written informed consent from the participants' legal guardian/next of kin was not required to participate in this study in accordance with the national legislation and the institutional requirements.

## Author Contributions

JS: conceptualization and formal analysis. JS and GH: writing—original draft. JS, TB, TF, BS, RH, and GH: feedback on analytical approach and writing—review and editing. All authors have made substantial contributions to the conception, the design of the work, and interpretation of results. All authors contributed to the article and approved the submitted version.

## Conflict of Interest

The authors declare that the research was conducted in the absence of any commercial or financial relationships that could be construed as a potential conflict of interest.

## Publisher's Note

All claims expressed in this article are solely those of the authors and do not necessarily represent those of their affiliated organizations, or those of the publisher, the editors and the reviewers. Any product that may be evaluated in this article, or claim that may be made by its manufacturer, is not guaranteed or endorsed by the publisher.
