# Supplementary material for: Lower Subjective Socioeconomic Status Is Associated With Increased Risk of Reporting Negative Experiences on Social Media. Findings From the “LifeOnSoMe”-Study
Source: Front Public Health. 2022 Jun 13;10:873463. doi: 10.3389/fpubh.2022.873463 (PMC9234458; doi:10.3389/fpubh.2022.873463)
Supplement: Supplementary file 1 [file Data_Sheet_1.docx]

Supplementary Material

**Supplementary table 1: Results from multinomial logistic regression across negative experiences on social media. Subjective socioeconomic status as independent variable (reference group; high SES (8-10)) and negative experiences (base category; never) on social media as dependent variable. Crude associations and adjusted for age and gender.**

| ***Variable*** | ***Crude,***  ***relative risk ratio (95%CI)*** | ***p-value*** | ***Adjusted,***  ***relative risk ratio (95%CI)*** | ***p-value*** |
| --- | --- | --- | --- | --- |
| **Unwanted attention from strangers (V1)** | Base category (Never) | N/A | Base category  (Never) | N/A |
| Seldom, low SES (0-4) | **1.72 (1.14-2.59)** | **0.009** | **1.51 (1.03-2.22)** | **0.034** |
| Sometimes or more, low SES (0-4) | **1.82 (1.25-2.67)** | **0.002** | 1.41 (0.98-2.02) | 0.064 |
| Seldom, medium SES (5-7) | 1.18 (0.96-1.45) | 0.108 | 1.09 (0.86-1.38) | 0.491 |
| Sometimes or more, medium SES (5-7) | **1.32 (1.14-1.54)** | **<0.001** | 1.15 (0.96-1.37) | 0.127 |
| **Others share pictures/videos (V2)** | Base category (Never) | N/A | Base category  (Never) | N/A |
| Seldom, low SES (0-4) | **1.78 (1.36-2.34)** | **<0.001** | **1.74 (1.30-2.32)** | **<0.001** |
| Sometimes or more, low SES (0-4) | 1.45 (0.90-2.33) | 0.129 | 1.47 (0.90-2.39) | 0.127 |
| Seldom, medium SES (5-7) | **1.44 (1.24-1.67)** | **<0.001** | **1.39 (1.20-1.62)** | **<0.001** |
| Sometimes or more, medium SES (5-7) | 1.17 (0.99-1.38) | 0.061 | 1.16 (0.96-1.40) | 0.135 |
| **Receive unwanted nude pictures/sexualised content (V3)** | Base category (Never) | N/A | Base category  (Never) | N/A |
| Seldom, low SES (0-4) | **1.68 (1.17-2.41)** | **0.005** | **1.50 (1.05-2.13)** | **0.025** |
| Sometimes or more, low SES (0-4) | **1.67 (1.17-2.38)** | **0.004** | **1.47 (1.03-2.11)** | **0.034** |
| Seldom, medium SES (5-7) | 1.10 (0.90-1.35) | 0.354 | 1.01 (0.82-1.25) | 0.913 |
| Sometimes or more, medium SES (5-7) | 1.13 (0.96-1.33) | 0.129 | 1.03 (0.86-1.22) | 0.753 |
| **Asked to send nude pictures/sexualised content (V4)** | Base category (Never) | N/A | Base category  (Never) | N/A |
| Seldom, low SES (0-4) | 1.15 (0.68-1.94) | 0.609 | 0.98 (0.58-1.67) | 0.951 |
| Sometimes or more, low SES (0-4) | **2.17 (1.54-3.04)** | **<0.001** | **1.80 (1.30-2.50)** | **<0.001** |
| Seldom, medium SES (5-7) | 1.10 (0.93-1.30) | 0.284 | 0.98 (0.81-1.19) | 0.853 |
| Sometimes or more, medium SES (5-7) | **1.44 (1.20-1.72)** | **<0.001** | **1.25 (1.00-1.57)** | **0.048** |
| **Negative comments (V5)** | Base category (Never) | N/A | Base category  (Never) | N/A |
| Seldom, low SES (0-4) | **2.24 (1.51-3.33)** | **<0.001** | **2.27 (1.53-3.38)** | **<0.001** |
| Sometimes or more, low SES (0-4) | **2.10 (1.19-3.71)** | **0.011** | **2.29 (1.27-4.16)** | **0.006** |
| Seldom, medium SES (5-7) | **1.38 (1.11-1.70)** | **0.003** | **1.35 (1.08-1.68)** | **0.008** |
| Sometimes or more, medium SES (5-7) | 1.01 (0.73-1.40) | 0.942 | 1.06 (0.77-1.47) | 0.719 |
| **Unpleasant/hurtful messages (V6)** | Base category (Never) | N/A | Base category  (Never) | N/A |
| Seldom, low SES (0-4) | **2.02 (1.45-2.83)** | **<0.001** | **2.02 (1.43-2.87)** | **<0.001** |
| Sometimes or more, low SES (0-4) | **2.56 (1.29-5.09)** | **0.007** | **2.54 (1.27-5.08)** | **0.008** |
| Seldom, medium SES (5-7) | **1.44 (1.09-1.89)** | **0.010** | **1.38 (1.04-1.84)** | **0.027** |
| Sometimes or more, medium SES (5-7) | 1.26 (1.00-1.60) | 0.054 | 1.24 (0.98-1.58) | 0.079 |
| **Negative talk from others (V7)** | Base category (Never) | N/A | Base category  (Never) | N/A |
| Seldom, low SES (0-4) | **1.88 (1.32-2.67)** | **<0.001** | **1.96 (1.40-2.75)** | **<0.001** |
| Sometimes or more, low SES (0-4) | **1.86 (1.03-3.35)** | **0.040** | **1.94 (1.04-3.62)** | **0.037** |
| Seldom, medium SES (5-7) | **1.44 (1.18-1.76)** | **<0.001** | **1.43 (1.18-1.73)** | **<0.001** |
| Sometimes or more, medium SES (5-7) | 1.06 (0.84-1.33) | 0.641 | 1.06 (0.83-1.35) | 0.636 |
| **Excluded from groups/chats (V8)** | Base category (Never) | N/A | Base category  (Never) | N/A |
| Seldom, low SES (0-4) | 1.28 (0.83-1.98) | 0.258 | 1.21 (0.78-1.89) | 0.392 |
| Sometimes or more, low SES (0-4) | **1.72 (1.08-2.76)** | **0.023** | 1.60 (0.97-2.63) | 0.066 |
| Seldom, medium SES (5-7) | **1.33 (1.09-1.62)** | **0.005** | **1.27 (1.05-1.55)** | **0.016** |
| Sometimes or more, medium SES (5-7) | **1.23 (1.04-1.45)** | **0.015** | 1.16 (0.99-1.35) | 0.073 |

95%CI: 95% Confidence interval; SES: Socioeconomic status. Bold indicates significant estimates.

Adjusted: Adjusted for age and gender.

Estimates based on multiple imputation while accounting for school-level clustering.

**Supplementary figure 1: Ridgeplots of the response distribution across socioeconomic status for A) Number of different negative experiences, B) Negative acts and exclusion, and C) Unwanted attention from others.**


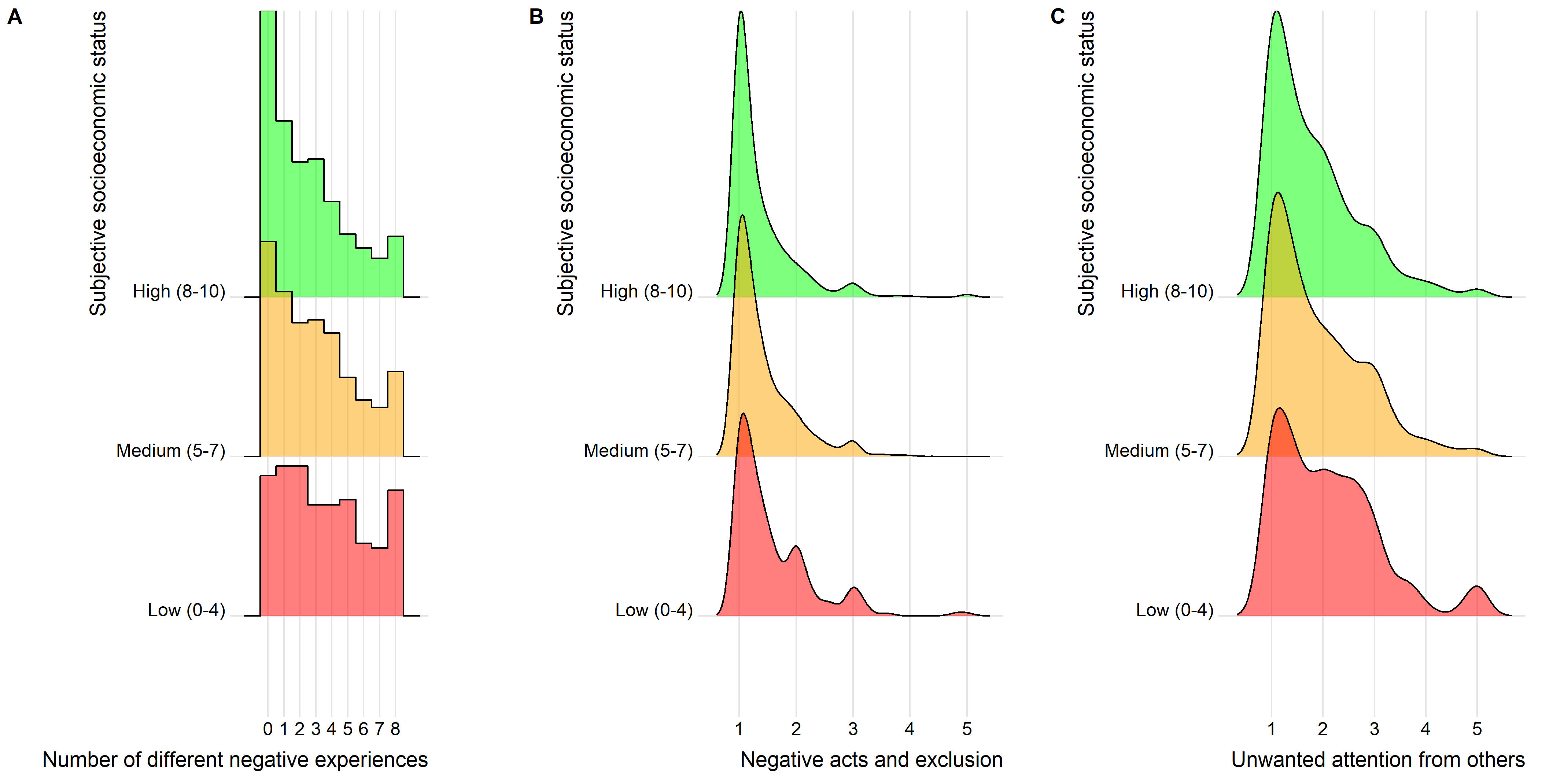


Probability that a random draw from low SES is larger than a random draw from high SES using Wilcoxon rank-sum tests: A) 62.1%, **p<0.001**; B) 56.7%, **p=0.001**; C) 55.6%, **p<0.001**

Probability that a random draw from low SES is larger than a random draw from medium SES using Wilcoxon rank-sum tests: A) 60.8%, **p<0.001**; B) 56.0%, **p=0.003**; C) 55.2%, **p<0.001**

Probability that a random draw from medium SES is larger than a random draw from high SES using Wilcoxon rank-sum tests: A) 59.3%, **p<0.001**; B) 56.1%, **p=0.004**; C) 53.1%, **p=0.003**

Distribution and Wilcoxon rank-sum tests based on non-imputed data (N ranging from 3415 to 3226). Clustering of data not accounted for.
